# Investigation of genetic diversity of Iranian wild relatives of bread wheat using ISSR and SSR markers

**DOI:** 10.1186/s43141-023-00526-5

**Published:** 2023-06-29

**Authors:** Maryam Jabari, Ahmadreza Golparvar, Behzad Sorkhilalehloo, Majid Shams

**Affiliations:** 1 Department of Agronomy and Plant Breeding, College of Agriculture, Islamic Azad University, Isfahan (Khorasgan) Isfahan Branch, Iran; 2 Genetic Research Department and the National Plant Genebank of Iran, Seed and Plant Improvement Institute, Agricultural Research, Education and Extension Organization, AREEO, Karaj, Iran

**Keywords:** Genetic diversity, DNA-based markers, *Aegilops*, *Triticum*, Polymorphism

## Abstract

**Background:**

Wild relatives of wheat are one of the most important genetic resources to use in wheat breeding programs. Therefore, identifying wild relatives of wheat and being aware of their diversity, is undeniably effective in expanding the richness of the gene pool and the genetic base of new cultivars and can be a useful tool for breeders in the future. The present study was performed to evaluate the molecular diversity among 49 accessions of the genera *Aegilops* and *Triticum* in the National Plant Gene Bank of Iran using two DNA-based markers, i.e., SSR and ISSR. Also, the present study aimed to examine the relationships among the accessions studied belonging to different genetic backgrounds.

**Results:**

Ten SSR and tan ISSR primers produced 2065 and 1524 polymorphism bands, respectively. The number of Polymorphic Bands (NPB), the Polymorphism Information Content (PIC), Marker Index (MI), and Resolving Power (Rp) in SSR marker was 162 to 317, 0.830 to 0.919, 1.326 to 3.167, and 3.169 to 5.692, respectively, and in the ISSR marker, it was from 103 to 185, 0.377 to 0.441, 0.660 to 1.151, and 3.169 to 5.693, respectively. This indicates the efficiency of both markers in detecting polymorphism among the accessions studied. The ISSR marker had a higher polymorphism rate, MI, and Rp than the SSR marker. Molecular analysis of variance for both DNA-based markers showed that the genetic variation within the species was more than the genetic diversity between them. The high level of genomic diversity discovered in the *Aegilops* and *Triticum* species proved to provide an ideal gene pool for discovering genes useful for wheat breeding. The accessions were classified into eight groups based on SSR and ISSR markers using the UPGMA method of cluster analysis. According to the cluster analysis results, despite similarities between the accessions of a given province, in most cases, the geographical pattern was not in accordance with that observed using the molecular clustering. Based on the coordinate analysis, neighboring groups showed the maximum similarities, and distant ones revealed the maximum genetic distance from each other. The genetic structure analysis successfully separated accessions for their ploidy levels.

**Conclusions:**

Both markers provided a comprehensive model of genetic diversity between Iranian accessions of *Aegilops* and *Triticum* genera. Primers used in the present study were effective, informative, and genome-specific which could be used in genome explanatory experiments.

## Background

Wheat is one of the most important staple foods in the world, which is ranked as second among various crops in terms of its area under cultivation and production rate [1]. This crop provides 20% of the total protein and calories needed for human nutrition as well as about 40% of essential micronutrients including zinc, iron, manganese, magnesium, and vitamins B and E for the majority of people whose diet depends on wheat [2]. Global demand for wheat is increasing along with population growth. The statistics show that the demand for wheat and its products will increase up to 50% by 2050 [3]. Producing more food to feed a growing population requires improvement of a more diverse genetic basis of breeding lines and access to new sources of genetic variation for breeding traits, e.g., yield components [4].

On the other hand, one of the most significant factors affecting wheat production is uncertainty in climate patterns [5]. In recent years, climate changes due to abiotic stresses such as drought, heat, and salinity have dramatically impacted the production of crops. Focusing on using new strategies to face the adverse conditions created by these changes and producing resistant/tolerant varieties for cultivation under such climate conditions is one of the basic requirements in breeding programs [6]. Productivity, flexibility, and sustainability of current agricultural systems are essential owing to the land limitation, water, and other inputs of natural resources, competition for arable land, soil degradation, and climate change. And the primary key to sustainable improvement is the use of genetic diversity in plant breeding for sustainable production [7, 8]. Using wild relatives can provide rich and diverse sources of new and ideal alleles for breeders considering the limitation of genetic diversity in modern important crop cultivars for the purpose of adaptation to climate change and other adverse environmental stresses, utilization of available allelic buffer in the gene pool of a given genetic resource germplasm can increase the chance of achieving new allelic diversity in these plant materials [9] Likewise, wheat germplasm is very important to overcome these challenges because native cultivars and wild relatives of wheat have untapped genetic diversity [10–12]. Indeed, native cultivars and their wild relatives contain the most appropriate genes due to their genetic capacity and adaptability to physiological conditions and adverse environmental factors, hence providing the genetic diversity required for plant breeders. Therefore, these genetic materials are considered valuable genetic resources, especially in resistance/tolerant to biotic and abiotic stresses, as well as in genetic improvement for other useful traits such as protein quality [13]**.** Various molecular marker techniques such as AFLP, RAPD, SSR, ISSR, and DArT have recently played a vital role in investigating diversity and evolutionary relationships [14]. The SSR markers are repetitive sequences consisting of one to six nucleotides distributed abundantly and uniformly in the coding and non-coding regions of the eukaryotes genome. These markers are widely used due to their multi-allelic buffering, codominant inheritance, and ease of molecular detection for its variability. The ISSR markers have been shown as efficient markers in genetic diversity studies because of their multilocus, dominance, repeatability, and high polymorphism features [15]. The SSR and ISSR markers have been applied to amplify orthologous sequences in phylogenetically related species in various plants, including wheat [16–18], durum wheat [19, 20], barley [21–23], triticale [24], sugar beet [25], oat [26], and maize [27].

Two genera *Aegilops* and *Triticum* belonging to the Poaceae family are considered the most important genetic resources of wheat. Iran is one of the richest centers of wheat diversity in the Middle East and the Fertile Crescent, so the existence of natural habitats of relative species of *Aegilops* and *Triticum* in these regions has led to the creation of one of the wealthiest wheat gene pools in the region [28]. The present study was conducted using SSR and ISSR markers to investigate genetic diversity and to study relationships among various accessions belonging to some species collected from different parts of Iran.

## Methods

### Plant material and DNA extraction

The plant materials studied in this experiment included 14 accessions of the genus *Triticum* and 35 accessions of the genus *Aegilops* from the National Plant Gene Bank of Iran (NPGBI). Control cultivars belonged to the *Triticum aestivum* which includes five cultivars of Mahoti, Kavir, Rakhshan, Talaei, and Torabi. Plant materials information, along with the genotype code and their geographical characteristics of the place of their collection, are presented in Table 1. The first control, Mahoti as native variety, was selected and introduced from the saline areas around Ardakan in Yazd under saline soil and irrigation conditions. The second control, Kavir, is a saline/drought-tolerant cultivar resulting from the crossing of a native Iranian cultivar resistant to an CIMMYT (International Wheat and Maize Breeding Research Center) line. Three other checks had also a genetic background from CIMMYT. Two checks, i.e., Talaei and Torabi, contain drought-resistance genes from *Aegilops tauschii*.

**Table 1:** List of the 54 investigated *Triticum* and *Aegilops* accessions

No.	Genebank Code	Species	Collection site	Ploidy level	Genome	Longitude	Latitude
1	Rakhshan	*T. aestivum*	Alborz	6	ABD	50° 94′ E	35° 78′ N
2	Kavir	*T. aestivum*	Alborz	6	ABD	50° 94′ E	35° 78′ N
3	Torabi	*T. aestivum*	Alborz	6	ABD	50° 94′ E	35° 78′ N
4	Mahoti	*T. aestivum*	Alborz	6	ABD	50° 94′ E	35° 78′ N
5	Talaei	*T. aestivum*	Alborz	6	ABD	50° 94′ E	35° 78′ N
6	KC-55059	*T. araraticum*	West Azerbaijan	4	AG	45° 47′ E	36° 15′ N
7	KC-30753	*T. boeoticum*	Alborz	2	AB	50° 75′ E	36° 18′ N
8	KC-55017	*T. boeoticum*	Kermanshah	2	AB	47° 14′ E	34° 32′ N
9	KC-30855	*T. boeoticum*	Hamadan	2	AB	48° 55′ E	34° 37′ N
10	KC-30796	*T. boeoticum*	Lorestan	2	AB	48° 08′ E	33° 54′ N
11	KC-55047	*T. boeoticum*	Kermanshah	2	AB	46° 52′ E	34° 14′ N
12	KC-30551	*T. boeoticum*	Uk22	2	AB		
13	KC-55001	*T. boeoticum*	Lorestan	2	AB	48° 77′ E	33° 90′ N
14	KC-30771	*T. boeoticum*	West Azerbaijan	2	AB	45° 48′ E	36° 25′ N
15	KC-30552	*T. boeoticum*	Uk01	2	AB		
16	KC-30532	*T. boeoticum*	Uk21	2	AB		
17	KC-29763	*A. biuncialis*	Kermanshah	4	UM	47° 09′ E	33° 37′ N
18	KC-29854	*A. columnaris*	Kurdistan	4	UM	46° 41′ E	36° 07′ N
19	KC-29848	*A. columnaris*	Kurdistan	4	UM	47° 08′ E	35° 19′ N
20	KC-28308	*A. columnaris*	West Azerbaijan	4	UM	45° 02′ E	37° 28′ N
21	KC-298541	*A. columnaris*	Kurdistan	4	UM	47° 31′ E	36° 13′ N
22	KC-29174	*A. crassa*	Hamadan	4	DM	48° 55′ E	34° 40′ N
23	KC-50021	*A. crassa*	Markazi	4	DM	49° 55′ E	34° 28′ N
24	KC-28348	*A. crassa*	Kermanshah	4	DM	46° 77′ E	33° 97′ N
25	KC-27754	*A. crassa*	West Azerbaijan	4	DM	45° 18′ E	37° 19′ N
26	KC-29021	*A. crassa*	Zanjan	4	DM	48° 33′ E	36° 56′ N
27	KC-29985	*A. umbellulata*	Qazvin	2	U	50° 29′ E	36° 26′ N
28	KC-28335	*A. umbellulata*	West Azerbaijan	2	U	44° 93′ E	37° 81′ N
29	KC-28321	*A. umbellulata*	West Azerbaijan	2	U	45° 03′ E	37° 21′ N
30	KC-28113	*A. umbellulata*	Hamadan	2	U	48° 14′ E	34° 11′ N
31	KC-29454	*A. triuncialis*	Gilan	6	UC	49° 50′ E	37° 31′ N
32	KC-29389	*A. triuncialis*	Semnan	6	UC	55° 00′ E	36° 47′ N
33	KC-28152	*A. triuncialis*	Fars	6	UC	51° 59′ E	30° 16′ N
34	KC-50074	*A. triuncialis*	West Azerbaijan	6	UC	45° 10′ E	38° 36′ N
35	KC-28128	*A. triuncialis*	Golestan	6	UC	55° 53′ E	37° 44′ N
36	KC-28992	*A. tauschii*	Kerman	2	D	56° 41′ E	29° 20′ N
37	KC-29692	*A. tauschii*	Qazvin	2	D	50° 24′ E	36° 26′ N
38	KC-29416	*A. tauschii*	Mazandaran	2	D	53° 27′ E	36° 41′ N
39	KC-29422	*A. tauschii*	Golestan	2	D	55° 11′ E	37° 05′ N
40	KC-28310	*A. tauschii*	West Azerbaijan	2	D	45° 09′ E	37° 20′ N
41	KC-28241	*A. tauschii*	Markazi	2	D	49° 20′ E	34° 37′ N
42	KC-29127	*A. tauschii*	Alborz	2	D	50° 96′ E	35° 08′ N
43	KC-29146	*A. cylindrica*	Isfahan	4	CD	51° 73′ E	32° 90′ N
44	KC-50085	*A. cylindrica*	East Azerbaijan	4	CD	47° 03′ E	39° 11′ N
45	KC-28367	*A. cylindrica*	Kermanshah	4	CD	47° 89′ E	34° 52′ N
46	KC-28325	*A. cylindrica*	West Azerbaijan	4	CD	44° 51′ E	37° 42′ N
47	KC-29128	*A. cylindrica*	Alborz	4	CD	50° 65′ E	35° 85′ N
48	KC-29145	*A. kotschyi*	Isfahan	4	US	52° 23′ E	32° 53′ N
49	KC-28956	*A. neglecta*	Kermanshah	4	UM	47° 32′ E	34° 47′ N
50	KC-28341	*A. neglecta*	East Azerbaijan	4	UM	47° 04′ E	37° 28′ N
51	KC-29430	*A. triuncialis*	Golestan	6	UC	55° 21′ E	36° 50′ N
52	KC-30523	*T. urartu*	Uk31	2	A		
53	KC-30905	*T. urartu*	Kermanshah	2	A	46° 40′ E	34° 42′ N
54	KC-55052	*T. urartu*	Kurdistan	2	A	46° 83′ E	35° 27′ N

*T Triticum, A Aegilops*

The studied accessions were cultivated in Coco peat and perlite substrate and kept in the growth room. Fresh leaf samples of the studied genotypes were collected during stages of 1–2 leaves of growth. Extracting genomic DNA of the desired samples was performed according to the Lodhi et al. [29] method using CTAB (Cethyl Trimethyl Ammonium Bromide) instructions. To ensure the accuracy of the extraction, the quantity and quality of DNA in each sample were measured using a Nanodrop device.

### Polymerase chain reaction using markers SSR and ISSR

Ten SSR primers and 10 ISSR primers were used to amplify the genomic DNA of the samples (Table 2). The polymerase chain reaction was prepared in a volume of 10 µl including 3 µl of deionized water, 1 µl of desired primer, 1 µl of Genomic DNA (50 ng concentration), and 5 µl of Master Mix 2X PCR. The polymerase chain reaction thermal cycle includes an initial denaturation step of 5 min at 94°C, followed by 35 cycles of 35 s at 94 °C, binding of 1-min 35 cycles primer at 44–60°C (depending on the type of primer). The duration of primer extension of 1-min 35 cycles, plus the final extension was 10 min. Primer extension of 1-min 35 cycles and the final extension performed at 72 centigrade. We used 1.5% agarose gel with 1X TAE buffer to separate and detect the amplified fragments. This stage is carried out by loading 6 µl of amplified DNA in each well with a voltage of 50 for 50 min. Safe view II was used for staining. Amplified fragments were then checked and detected with a Gel Document device.

**Table 2:** Characteristics of SSR and ISSR primers and results of genetic study of genetic accessions of wheat relatives and experimental controls

Primer	Locus	Primer sequence	NB	NPB	PIC	MI	Rp	Synth. (bp)	Source
SSR									
SSR-1	Xgwm469-6D	L-CAACTCAGTGCTCACACAACG R-CGATAACCACTCATCCACACC	211	8	0.849	1.947	4.857	170	Roder et al., 1998 [30]
SSR-2	Xgwm484-2D	L-ACATCGCTCTTCACAAACCC R-AGTTCCGGTCATGGCTAGG	243	12	0.893	2.359	5.283	143	Roder et al., 1998 [30]
SSR-3	Xgwm608-4D	L-ACATTGTGTGTGCGGCC R-GATCCCTCTCCGCTAGAAGC	253	10	0.878	2.415	5.500	144	Roder et al., 1998 [30]
SSR-4	Xgwm33-1D	L-GGAGTCACACTTGTTTGTGCA R-CACTGCACACCTAACTACCTGC	147	7	0.830	1.326	3.196	158	Roder et al., 1998 [30]
SSR-5	Xgwm106-1D	L-CTGTTCTTGCGTGGCATTAA R-AATAAGGACACAATTGGGATGG	162	9	0.861	1.516	3.522	81	Roder et al., 1998 [30]
SSR-6	Xgwm192-5D	L-GGTTTTCTTTCAGATTGCGC R-CGTTGTCTAATCTTGCCTTGC	317	14	0.919	3.167	6.891	232	Roder et al., 1998 [30]
SSR-7	Xgwm210-2D	L-TGCATCAAGAATAGTGTGGAAG R-TGAGAGGAAGGCTCACACCT	298	11	0.886	2.870	6.478	182	Roder et al., 1998 [30]
SSR-8	Xgwm292-5D	L-TCACCGTGGTCACCGAC R-CCACCGAGCCGATAATGTAC	162	8	0.862	1.518	3.522	188	Roder et al., 1998 [30]
SSR-9	Xgwm295-7D	L-GTGAAGCAGACCCACAACAC R-GACGGCTGCGACGTAGAG	120	6	0.816	1.064	2.609	258	Roder et al., 1998 [30]
SSR-10	Xgwm314-3D	L-AGGAGCTCCTCTGTGCCAC R-TTCGGACTCTCTTCCCTG	152	7	0.823	1.360	3.304	171	Roder et al., 1998 [30]
Mean			206	9.2	0.862	1.954	4.489		
ISSR									
UBC-813		CTCTCTCTCTCTCTCTT	150	5	0.415	0.957	4.615		Fathi et al., 2014 [31]
UBC-857		ACACACACACACACACAT	167	7	0.441	1.134	5.138		Hajiyev et al., 2015 [32]
M-9		CGTGGGTGGGTG	185	7	0.404	1.151	5.692		
UBC-840		GAGAGAGAGAGAGAGAT	103	5	0.417	0.660	3.169		Khodaee et al., 2021 [33]
UBC-825		ACACACACACACACACT	171	7	0.431	1.133	5.262		Fathi et al., 2014 [31]
ISSR-3		TGAACACACACACACACA	179	8	0.404	1.111	5.508		
UBC-815		CTCTCTCTCTCTCTCTG	115	5	0.415	0.735	3.538		Patill et al., 2013 [34]
UBC-811		GAGAGAGAGAGAGAGAC	149	7	0.393	0.901	4.585		Thomas and Bebeli, 2010 [35]
M-7		ATGATGATGATGATGATG	162	7	0.377	0.939	4.985		Etminan et al., 2018 [28]
UBC-880		GGAGAGGAGGAGAGGAGA	143	6	0.407	0.896	4.400		Bouziani et al., 2019 [36]
Mean			152	6.5	0.410	0.962	4.689		

*PIC* polymorphism information content, *NB* number of bands, *NPB* number of polymorphic bands, *n* number of bands per genotype, *MI* marker index, *Rp* resolving power

### Data analysis

The allelic banding patterns was scored as zero and one (as a binary system) for the absence and presence of a band pattern, respectively. Then, various indicators such as polymorphism information content (PIC), resolving power (RP), marker index (MI), total amplified bands (NTB), and number of polymorphic amplified bands (NPB) were used to check and compare the efficiency of various molecular markers and primers. The calculation relationships of each of these indicators are given below:

1 $$\mathrm{PIC }= 1 -\mathrm{ \Sigma \textit{P}i^2}$$

2 $$\mathrm{Rp }=\mathrm{ \Sigma I_{b}},\;\mathrm{ I_{b} }= 1 - [2\times (0.5-{\mathrm{p}}_{\mathrm{i}})]$$

3 $$\mathrm{MI }=\mathrm{ PIC }\times \mathrm{\; EMR}, \mathrm{ EMR }={\mathrm{N}_{\text{p}}(\mathrm{N}_{\text{p}}/\mathrm{N})^4}$$

These relationships, pi, Np, and N, respectively, indicate the allele frequency, the number of polymorphic bands, and the total number of amplified bands from one primer [37].

The use of molecular markers in this research aimed to depict the genetic diversity among the accessions studied in terms of differences attributed to the genus and species, ploidy level, and genomic formula of wheat relatives and the control checks. In order to clarify the relationship between the phenotypic and genotypic data and to select suitable parents for wheat pre-breeding programs, the phenotypic and genotypic diversity of the accessions were compared to each other. Also, the differences within and between the studied populations were performed through molecular variance analysis to explain the differences attributed to the genus and species, ploidy level, and genomic formula of wheat relatives and the check cultivars in this experiment. The genetic structures of 54 genetic samples of *Triticum* and *Aegilops* were analyzed using STRUCTURE an Genalex softwares.

## Results

### Molecular analysis of markers

**Fig. 1:**
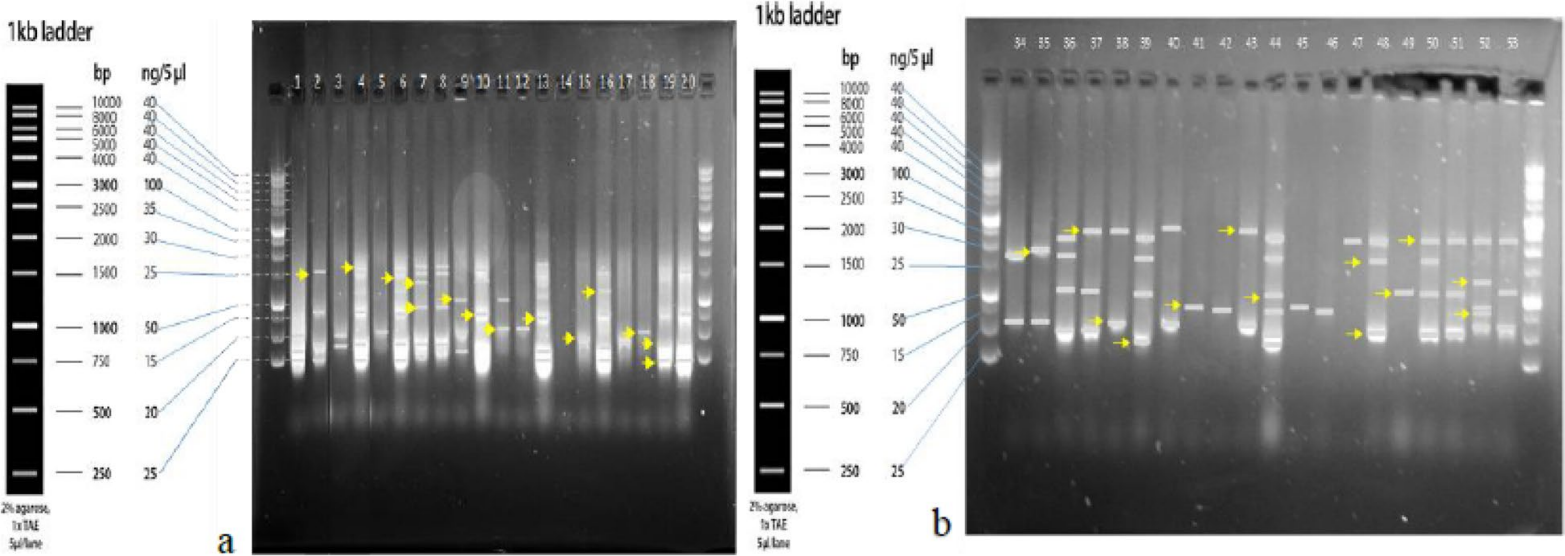
Scoring of the bands resulting from the marker ISSR-M9 **a** and marker SSR-Xgwm192-5D **b** Genotype status of wheat relatives of National Plant Gene Bank of Iran

Two examples of the scoring method of the genotypes of wheat relatives of NPGBI after revealing the bands obtained from the markers are shown in Fig. 1. For instance, in the gel “a”, the left and right columns allocated to the 1 kbp Ladder where the position of multi-shape (polymorphic) bands of marker 9 has been demonstrated, and the middle columns were the accessions of NPGBI.

All of the ISSR and SSR primers produced repeatable polymorphic bands. For the ISSR marker, the total produced bands were 1524 bands. The number of polymorphism bands varied from 103 (UBC-840 primer) to 185 (M9 primer), while 152 bands were the average number of observed bands. For each accession, the average number of bands and polymorphism were estimated at 2.345 and 100%, respectively. The average amount of polymorphism information content (PIC), the highest amount of polymorphism, and the lowest amount of polymorphism were 0.41, 0.441 (UBC-857 primer), and 0.377 (M-7 primer), respectively. The highest and lowest index of MI markers were observed in the M-9 (0.660) and UBC- 857 (1.151), respectively. The resolving power of primers (Rp) differed from 3.169 (UBC-840 primer) to 5.692 (M-9 primer). However, its average amount was 4.689.

For SSR markers, the number of total produced bands, average observed bands, and polymorphism were 2065, 206, and 59%, respectively. The number of bands varied from 120 (Xgwm295-7D primer) to 317 (Xgwm192-5D). The average number of bands for each genotype was reported as 2.245 on average. The percentage of polymorphic bands produced by all primers was 100%. The average amount of polymorphism information content (PIC), marker index (MI), and resolving power of primers (Rp) were 0.862, 1.954, and 4.489, respectively. The highest amount was for Xgwm192-5D primer with 317, 3.167, and 6.891 for three parameters of PIC, MI, and Rp. On the other hand, the lowest amount was for Xgwm295-7D primer with 120, 1.064, and 2.609 for three parameters of PIC, MI, and Rp. In general, applied ISSR and SSR primers statistics illustrated the power of the markers in explaining the molecular variations among the genotypes studied.

### Genetic diversity among the species

The results of the MANOVA analysis are presented in Table 3. Based on the results, the variance within the populations is very high and includes 74.1, 85.7, and 91.1% of the total variance, respectively. Also, the variance among the populations was significant; it indicated significant genetic differences among the accessions of the groups attributed to species, genomic, and ploidy classification, in terms of polymorphic bands.

**Table 3:** Analysis of molecular variance (AMOVA) of the accessions of the wheat relatives and controls usage of the ISSR and SSR markers data for the species (a), genome (b) and ploidy groups (c)

	Sources of variation	df	Sum of squares	Mean square	Variance percentage	P hi
a	Between populations	9	643.2	71.5	25.9	0.260 ^**^
	Within populations	44	1100.2	25.0	74.1	
	Total	53	1743.4		100.0	
b	Between populations	3	277.0	92.3	14.3	0.143 ^**^
	Within populations	53	1466.5	29.3	85.7	
	Total	56	1743.426		100.0	
c	Between populations	2	157.1	78.8	8.9	0.089 ^**^
	Within populations	51	1586.4	31.1	91.1	
	Total	53	1743.5		100.0	

^*^and ^**^significant at 5 and 1% probability levels, respectively

The differentiation between populations (Fst-value) average was high for both ISSR and SSR marker systems (Table 4) which a confirmatory for AMOVA results.

**Table 4:** Fst average values for 54 accessions of wheat wild relatives based on ISSR and SSR markers

Marker	Subpopulation			Mean
	1	2	3	
ISSR	0.7423	0.5387	0.4375	0.5728
SSR	0.2860	0.6154	0.7322	0.5445
ISSR-SSR	0.7705	0.5857	0.3945	0.5836

### The revealed genetic diversity using molecular markers

The statistics obtained from Genalex software were used to explain the situation of the genetic distance of accessions attributed to climatic groups. Estimated genetic alteration parameters, including the average number of observed alleles, the number of effective alleles, Shannon information index, expected heterozygosity, and polymorphism percentage are presented in Table 5. Based on the data of both SSR and ISSR, at the level of species, the highest number of effective allele, Shannon index, and polymorphism percentage were related to *aestivum* species (0.46, 1.51, and 85%, respectively). Also, at the genome level, it was related to the ABD genome (0.461, 1.508, and 85%, respectively), and at the ploidy level, it was related to the 2n=4X=28 ploidy level (0.479, 1.532, and 93%, respectively). After *aestivum*, the highest diversity was for the *boeoticum* and *Tauschii* species. Moreover, the lowest diversity was observed for the *neglecta* species, U genome, and 2n=2X=14 ploidy level.

**Table 5:** Genetic characteristics of wheat relatives’ accessions using ISSR and SSR markers data used for the grouping of species (a), genome (b), and ploidy level (c)

(a)																		
Species		Number of accessions	Number of observed alleles	Number of effective alleles		Shannon’s information Index		Expected heterozygosity	Unbiased expected heterozygosity			Expected heterozygosity			Polymorphism percentage	Band Freq.	Allele frequency	
																	*p*	*q*
*aestivum*		5	1.8	1.51		0.46		0.34	0.34			0.34			0.85	0.48	0.32	0.68
*boeoticum*		10	1.69	1.48		0.42		0.29	0.29			0.29			0.81	0.46	0.32	0.68
*columnaris*		4	1.26	1.39		0.33		0.25	0.25			0.25			0.59	0.38	0.27	0.73
*crassa*		5	1.23	1.28		0.26		0.19	0.19			0.19			0.52	0.39	0.31	0.69
*umbellulata*		4	1.16	1.29		0.25		0.19	0.19			0.19			0.43	0.51	0.43	0.57
*triancialis*		8	1.61	1.33		0.33		0.23	0.23			0.23			0.75	0.37	0.27	0.73
*tauschii*		7	1.68	1.52		0.43		0.32	0.32			0.32			0.78	0.53	0.38	0.62
*cylindrica*		5	1.13	1.29		0.27		0.19	0.19			0.19			0.52	0.32	0.23	0.77
*neglecta*		2	0.52	1.13		0.11		0.10	0.10			0.10			0.18	0.25	0.22	0.78
*urartu*		4	1.13	1.40		0.31		0.25	0.25			0.25			0.52	0.39	0.28	0.72
**Total**		**54**	**1.32**	**1.36**		**0.32**		**0.24**	**0.24**			**0.24**			**0.59**	**0.41**	**0.30**	**0.70**
Species		Number of accessions	Number of observed alleles			Number of effective alleles			Shannon Diversity Index				Expected heterozygosity			Polymorphism percentage		
			Mean	Standard deviation		Mean		Standard deviation	Mean		Standard deviation		Mean		Standard deviation			
*aestivum*		5	1.8	0.02		1.51		0.02	0.46		0.02		0.34		0.01	0.85		
*boeoticum*		10	1.69	0.02		1.48		0.03	0.42		0.02		0.29		0.02	0.81		
*columnaris*		4	1.26	0.02		1.39		0.03	0.33		0.02		0.25		0.02	0.59		
*crassa*		5	1.23	0.02		1.28		0.03	0.26		0.02		0.19		0.02	0.52		
*umbellulata*		4	1.16	0.02		1.29		0.03	0.25		0.02		0.19		0.02	0.43		
*triancialis*		8	1.61	0.02		1.33		0.02	0.33		0.02		0.23		0.01	0.75		
*tauschii*		7	1.68	0.02		1.52		0.03	0.43		0.02		0.32		0.02	0.78		
*cylindrica*		5	1.13	0.02		1.29		0.03	0.27		0.02		0.19		0.02	0.52		
*neglecta*		2	0.52	0.02		1.13		0.02	0.11		0.02		0.10		0.02	0.18		
*urartu*		4	1.13	0.03		1.40		0.03	0.31		0.03		0.25		0.02	0.52		
Total		54	1.32	0.02		1.36		0.03	0.32		0.02		0.24		0.02	0.59		
(b)																		
Genome	Number of accessions		Number of observed alleles	Number of effective alleles		Shannon’s information Index	Expected heterozygosity		Unbiased expected heterozygosity		Polymorphism percentage		Band Freq.		Allele frequency			
															p	q		
ABD	5		1.803	1.508		0.461	0.341		0.34		0.85		0.48		0.32	0.68		
A	14		1.803	1.506		0.441	0.304		0.30		0.88		0.44		0.29	0.71		
U	18		1.783	1.394		0.387	0.256		0.26		0.83		0.41		0.29	0.71		
D	17		1.834	1.478		0.457	0.308		0.31		0.90		0.40		0.25	0.75		
**Total**	**54**		**1.81**	**1.47**		**0.44**	**0.30**		**0.30**		**0.86**		**0.43**		**0.29**	**0.71**		
Genome	Number of accessions		Number of observed alleles			Number of effective alleles			Shannon Diversity Index		Expected heterozygosity		Polymorphism percentage					
			Mean		Standard deviation	Mean	Standard deviation		Mean	Standard deviation	Mean		Standard deviation					
ABD	5		1.803		0.040	1.508	0.023		0.461	0.017	0.341		0.014		0.85			
A	14		1.803		0.045	1.506	0.029		0.441	0.019	0.304		0.015		0.88			
U	18		1.783		0.042	1.394	0.024		0.387	0.018	0.256		0.013		0.83			
D	17		1.834		0.041	1.478	0.022		0.457	0.015	0.308		0.011		0.90			
Total	54		1.81		0.040	1.47	0.02		0.44	0.02	0.30		0.01		0.86			
(c)																		
Genome	Number of accessions		Number of observed alleles		Number of effective alleles	Shannon’s information Index	Expected heterozygosity		Unbiased expected heterozygosity	Polymorphism percentage				Band Freq.	Allele frequency			
															*p*	*q*		
*2n=6X=42*	5		1.803		1.508	0.461	0.31		0.34	0.85				0.48	0.32	0.68		
*2n=4X=28*	25		1.885		1.532	0.479	0.32		0.32	0.93				0.44	0.28	0.72		
*2n=6X=42*	24		1.873		1.463	0.452	0.29		0.30	0.92				0.40	0.25	0.75		
**Total**	**54**		**1.85**		**1.50**	**0.46**	**0.30**		**0.32**	**0.90**				**0.44**	**0.28**	**0.72**		
Ploidy level	Number of accessions		Number of observed alleles			Number of effective alleles			Shannon Diversity Index	Expected heterozygosity					Polymorphism percentage			
			Mean		Standard deviation	Mean	Standard deviation		Mean	Standard deviation				Mean	Standard deviation			
*2n=6X=42*	5		1.803		0.040	1.508	0.023		0.461	0.014				0.341	0.014	0.85		
*2n=4X=28*	25		1.885		0.035	1.532	0.025		0.479	0.012				0.324	0.012	0.93		
*2n=2X=14*	24		1.873		0.036	1.463	0.021		0.452	0.011				0.299	0.011	0.92		
Total	54		1.85		0.040	1.50	0.02		0.46	0.01				0.32	0.01	0.90		

For a better understanding of the similarities and differences in accessions, the Nei similarity coefficient matrix for NPGBI accessions was calculated using ISSR and SSR markers as presented in Table 6. The similarity coefficient among the groups varied from 0.695 (for *neglecta* and *umbellulate* species) to 0.956 (for *triuncialis* and *crassa* species).

**Table 6:** Nei similarity coefficient matrix for the populations of wheat relatives use of markers ISSR and SSR for the groups of species (a), genome (b), and ploidy (c)

A	Species	*aest*	*boet*	*colm*	*cras*	*umbl*	*trun*	*tchi*	*cyln*	*nglt*	*urar*
	Aestivum *(aest)*	1.000									
	*Boeoticum (boet)*	0.821	1.000								
	*Columnaris (colm)*	0.855	0.923	1.000							
	*Crassa (cras)*	0.800	0.802	0.925	1.000						
	*Umbellulata (umbl)*	0.710	0.751	0.757	0.800	1.000					
	*Triuncialis (trun)*	0.839	0.856	0.932	0.956	0.855	1.000				
	*Tauschii (tchi)*	0.804	0.818	0.861	0.879	0.777	0.912	1.000			
	*Cylindrical (cyln)*	0.875	0.854	0.924	0.877	0.740	0.901	0.848	1.000		
	*Neglecta (nglt)*	0.829	0.815	0.895	0.877	0.695	0.879	0.800	0.907	1.000	
	*urartu (urar)*	0.841	0.905	0.891	0.818	0.729	0.850	0.809	0.865	0.888	1.000
B	Genome	ABD	A	U	D						
	ABD	1.000									
	A	0.854	1.000								
	U	0.837	0.898	1.000							
	D	0.899	0.941	0.936	1.000						
C	Ploidy Level	6X	4X	2X							
	*2n=6X=42*	1.000									
	*2n=4X=28*	0.882	1.000								
	*2n=2x=14*	0.895	0.975	1.000							

At the levels of genome and ploidy, the Nei similarity coefficient was different from 0.837 (for U and A genomes) to 0.936 (for D and U genomes), and from 0.882 (for 4X and 6X ploidy levels) to 0.975 (for 2x and 4X ploidy level), respectively.

### Cluster analysis

**Fig. 2:**
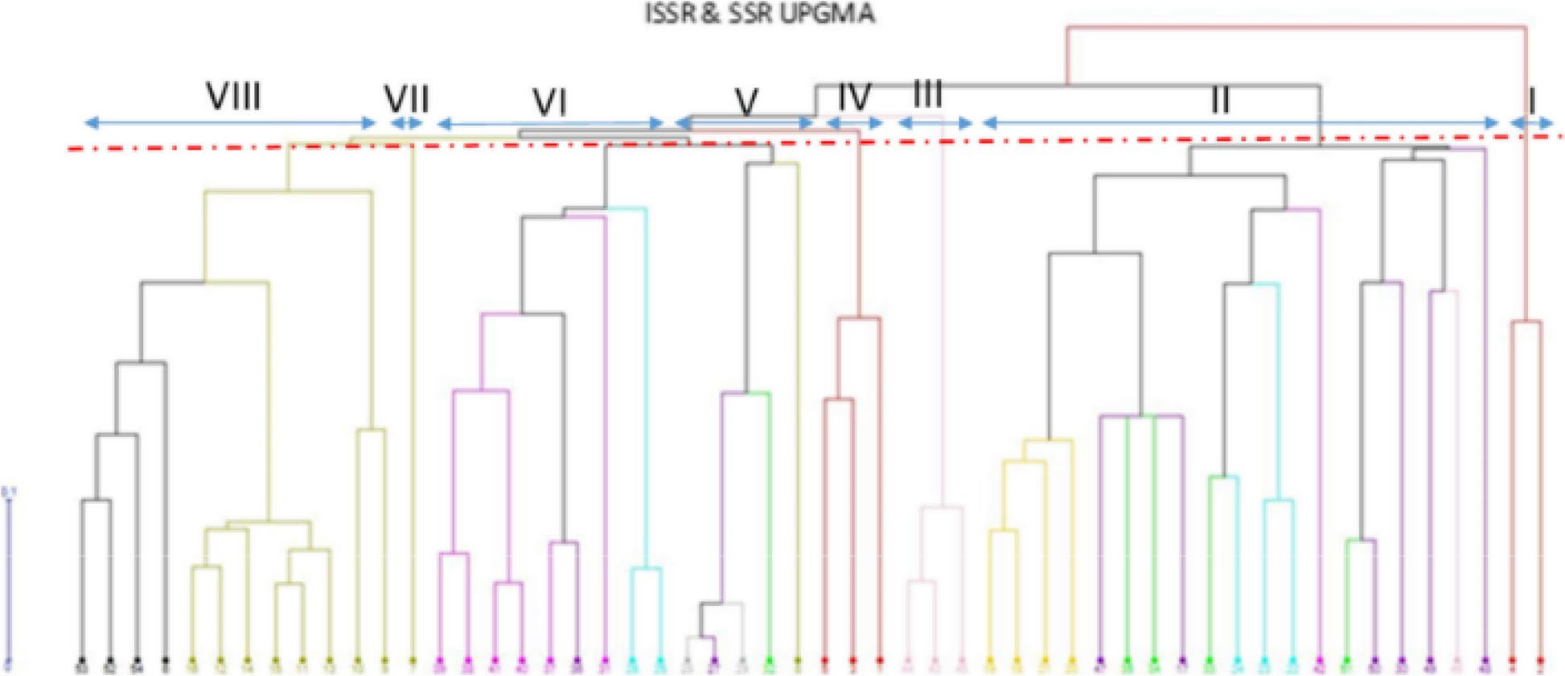
Cluster analysis of markers ISSR and SSR for the wheat relatives and experimental controls by the method UPGMA

**Fig. 3:**
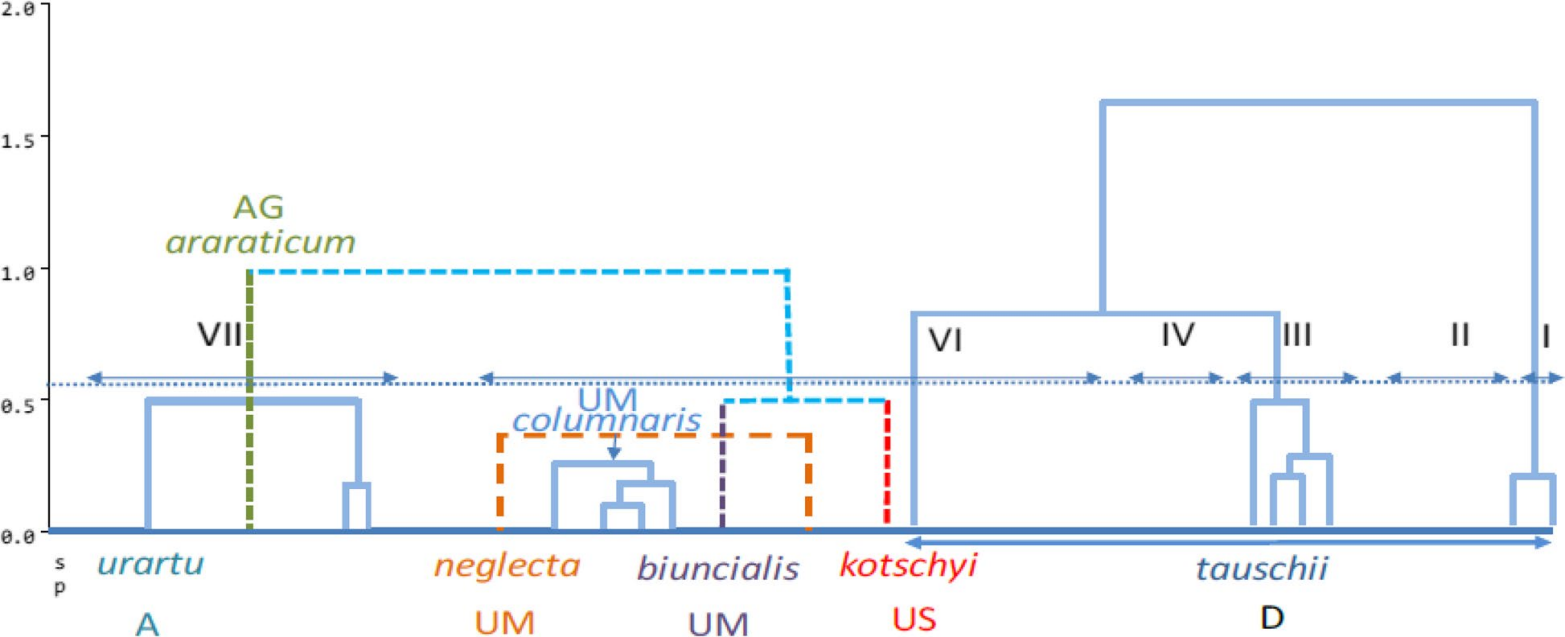
Grouping using cluster analysis of wild relatives of wheat of National Plant Gene Bank of Iran

The cluster analysis was conducted by the UPGMA method and Jaccard similarity coefficient. According to the data from both SSR and ISSR markers, all accessions were placed in the eight main groups, which in cluster 1, Mahoti and Kavir controls were grouped together. Cluster 2 consisted of 19 accessions belonging to nine species. Cluster 3 included three accessions of *cylindrica* species. Three controls of *aestivum* species, namely Rakhshan, Talai, and Tarabi, were included in cluster 4. Five accessions of three species *triuncialis*, *umbellulata*, and *boeoticum* were included in cluster 5. Cluster 6 consisted of nine species belonging to the genus *Aegilops*, namely six *tauschii* species, two *crassa* species and one *triuncialis* species. Cluster 7 alone contained one *boeoticum* species. Cluster 8 included 12 genes belonging to the genus *Triticum*, including eight accessions of *boeoticum* species, three accessions of *urartu* species and one accessions of *araraticum* species (Fig. 2). The species of *tauschii* with the origin of Gazvin and Kermanshah had the maximum genetic distances (Fig. 3). As demonstrated in the dendrogram of genotypic data, genotypes, like the phenotypic clusters, showed a vast genetic diversity. By carefully studying each cluster’s members, it can be easily observed that in most cases, the molecular pattern was not in accordance with the geographical distribution of accessions, despite the similarities among the accessions of a given province. Some of the genotypes, like quantitative data analysis, were placed at a distance from other members of a similar cluster due to polymorphic band differences. The Garmsar accession, with minimum polymorphic bands, was placed alone in one cluster.

Genetic distance between populations and sub-populations of wheat wild relatives and membership of accessions is presented in Table 7. The maximum and minimum genetic distance observed between A and C, and B and C groups, respectively. The highest number of accessions belonged to the C3 sub-group.

**Table 7:** Genetic distance between populations and sub-populations estimated using UPGMA method based on ISSR-SSR data

Population			Sub-population				
A	B		B1	B2	C1	C2	
66.250		B	64.583				B2
67.317	67.210	C	67.407	73.250			B3
					62.567		C2
					66.738	62.551	C3
A	^£^32, 29, 27, 28						
B1	8, 22, 23						
B2	30, 50, 51, 46, 49, 48, 24, 33						
B3	42, 35, 34, 17, 47, 21, 20, 18, 19						
C1	2, 4, 45, 43, 44						
C2	9, 10, 13, 11, 15, 14, 12, 16, 6, 54, 52, 53						
C3	1, 3, 5, 7, 25, 26, 31, 36, 37, 40, 41, 38, 39						

^£^ indicates the membership of each accession

### Principal coordinates analysis (PCoA)

**Fig. 4:**
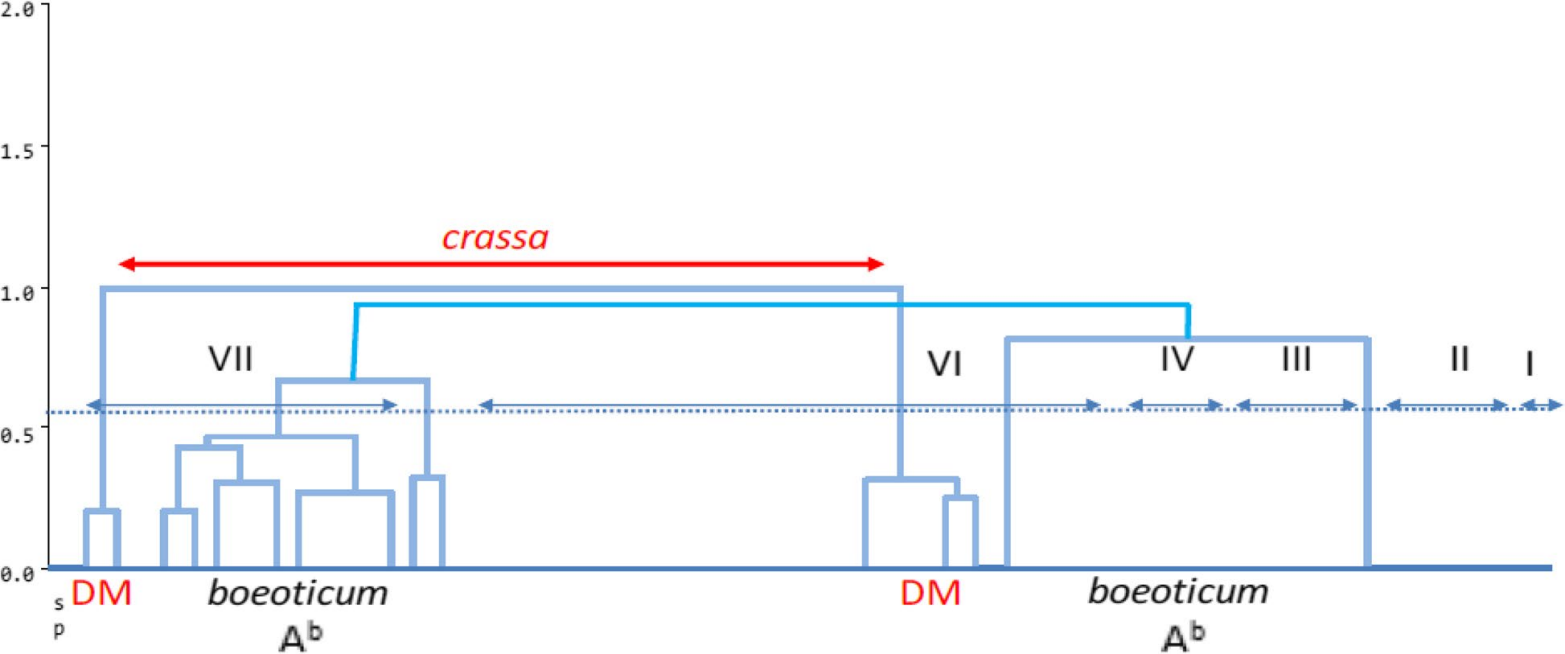
The distribution of different wheat species using ISSR and SSR markers based on coordinate analysis

**Fig. 5:**
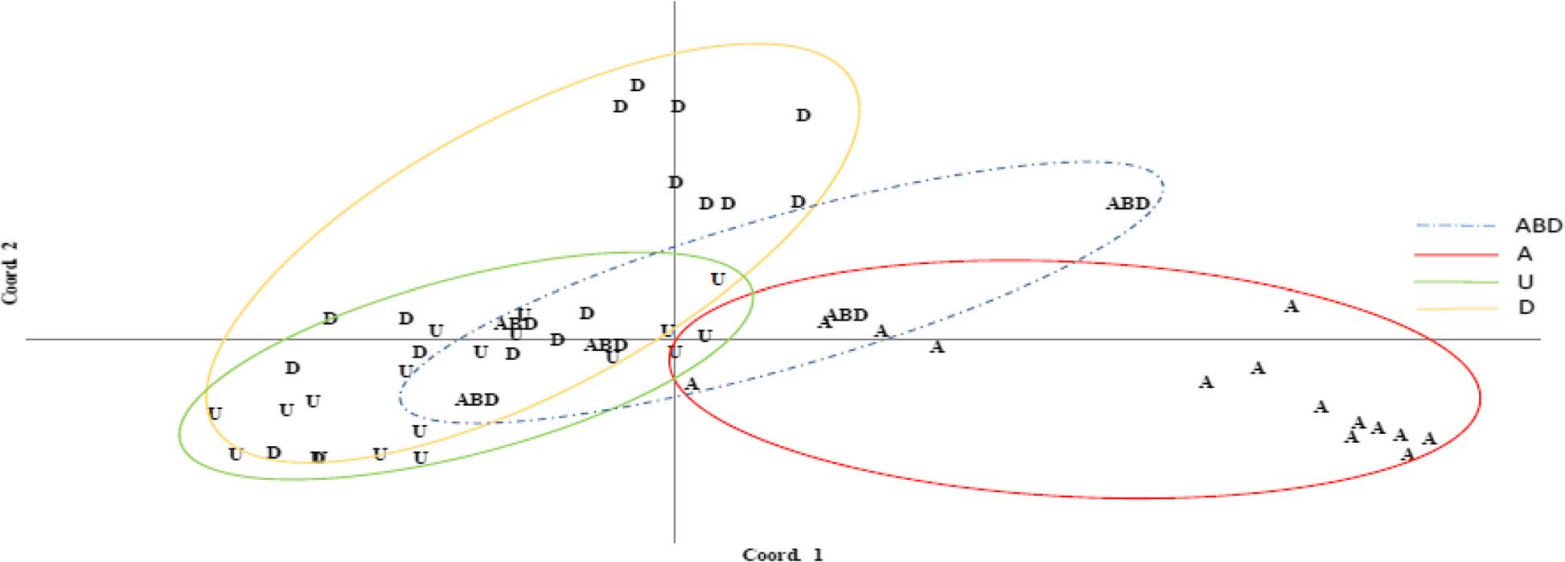
The distribution of different wheat relative genome using ISSR and SSR markers based on coordinate analysis

To understand the similarities and differences of groups formed based on species and genome formula, coordinate analyses were used, and Figs. 4 and 5 refer to the grouping of the accessions of NPGBI investigated, respectively. In principle coordinates analysis, the two first components explained 76 and 19 percent of the total variation, respectively. As has been found in Fig. 4, the highest genetic similarity is for neighboring groups, while the highest genetic distance is for distant groups.

As shown in Fig. 4, the maximum amount of within species variabilities were observed for the *boeticum*, *aestivum*, and *triuncialis* species, respectively, whereas the minimum levels of within variabilities were observed for the *tauschii*, *cylindrica*, and *columnaris* species, respectively. The *neglecta* species had the lowest within-group variability; however, its record did not take into account because it only had two samples studied. The most distant accessions belonged to the *boeoticum* and *triuncialis* species, whereas the most similar accessions found to be for the species, *cylindrica* and *columnaris*. The accessions of *urartu* with *boeoticum* as well as those of *triuncialis* with *crassa*, *columnaris*, and *cylindrica*, and *aestivum* with *boeoticum*, *triuncialis*, and *cylindrica* demonstrated the highest rate of between-species similarities. Interestingly, *tauschii* accession had no similarity with other groups except for some *crassa* accessions.

The A and D genomes had the maximum and the U genome had the minimum variability proportion (Fig. 5). The D genome had the highest similarity with the U genome while did not have any commonality with the A genome. The maximum genetic distance observed between accessions of A and D genomes. The U genome had commonality with all of the genomes studied. The ABD genome (bread wheat) which developed from its wild relatives through evolutionary prosses had a commonality with all others and located in the center of the plot. Additionally, the ABD genome showed high similarity with U and D genomes. The genome D had a wide distribution across both the first and second coordinates.

### Structure analysis

**Fig. 6:**
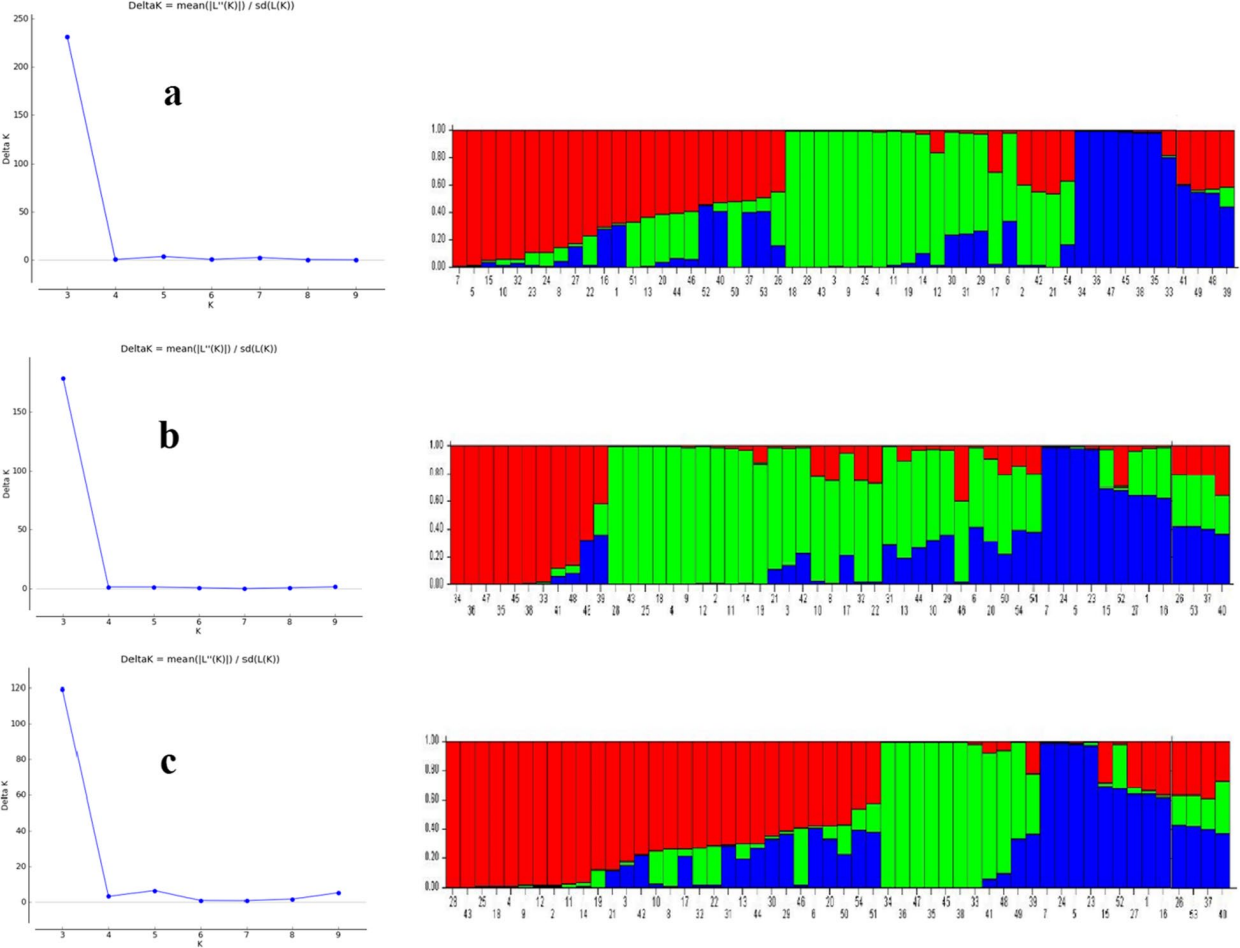
The genetic structure analysis in wheat wild relatives using ISSR **a**, SSR **b**, and ISSR-SSR **c** data. K value reflects the number of subpopulations. Red, green, and blue bars represent the membership coefficients of accessions based on allele frequencies for sub-populations

The genetic structures of 54 accessions of *Triticum* and *Aegilops* were performed and three groups were identified based on maximum-likelihood method (Fig. 6). The ISSR, SSR primers, and both together divided accessions into three groups. These groups correspond to the A, D, and U genomes in wheat wild relatives. Selection for genome A was properly done and all accessions of this genome belonged in a group.

## Discussion

In recent years, climate changes have seriously affected agricultural production due to abiotic stresses, such as drought, heat, and salt [38]. These alterations are seriously threatening the productivity of the cultivated wheat [39]. Wheat improvers need to access new genetic resources to support the growing human population demands and to produce more food supplies with higher quality in variable environmental conditions [38]. Wild relatives of wheat are precious resources for useful genetic diversity, which can be used for improving the crops such as wheat [40, 41]. *Triticum* and *Aegilops* genomes are the principal parts of the domesticated wheat genetic repository, which contains various species with diverse genomes. The studies revealed that each of the wild relatives has useful alleles, which can be applied in different environmental conditions for cultivated wheat [27]. Therefore, evaluating the genetic diversity within and between the bread wheat wild relatives is necessary before assessing the resistance to biotic and abiotic stresses [33]. Genetic diversity analysis through DNA-based molecular markers has been used as an efficient approach for estimating genome diversity and population structure to utilize in different plants.

In the present research, SSR and ISSR markers were used to investigate within and between species genetic diversity in *Aegilops*, *Triticum*, and five bread wheat varieties as control checks. Previously, Nouri et al. [42] used the ISSR marker to evaluate the polymorphism in accessions of *Aegilops* species. Moreover, Pour-Aboughadareh et al. [43] used SSR marker to investigate the polymorphism in accessions of *Aegilops* and *Triticum* species. The efficiency of these markers for designating the polymorphism had been approved. ISSR and SSR primers can target the microsatellites, which can be found plentifully in the plant genome, hence more repeatable markers, as compared to the other markers, e.g., RAPD [44]. Resolution power (Rp) and the polymorphism information content (PIC), in addition to polymorphism percentage, are essential factors of the usefulness of the marker, which are used to compare the efficiency of the marker for genetic analysis. Botstein et al. [45] concluded that primers with 0.25 to 0.50% PIC containbeneficial information for genetic diversity studies. In the present study, the PIC values ranged from 0.377 to 0.919. The average PIC values for ISSR (0.410) and SSR (0.862) indicated the effectiveness of applied primers to demonstrate genetic diversity analysis as well as grouping the accessions of different *Aegilops* and *Triticum* species (Fig. 2).

Higher values of MI and Rp for SSR primers validate more clarity and powers of these primers as compared to ISSR primers. Etminan et al. [19] used ISSR and SCoT to detect the genetic diversity in durum wheat genotypes. They concluded that MI and Rp could be the most important indices for determining the effectiveness of a marker. Therefore, ISSR primers proved to be more effective as compared to the SCoT primers. In contrast, Shaban et al. [46] investigated the efficiency of two ISSR and SCoT markers; based on polymorphism percentage, Rp, and MI parameters, SCoT primers were more effective than ISSR primers. Cluster analysis results based on three SCoT, CBDP, and SSR markers, and combined data, showed that the marker of SSR has more efficiency in grouping of *Ae. Tauschii*, *Ae. cylindrica*, *Ae. crassa*, and *T. aestivum* in two *Aegilops* and *Triticum* species [43]. Also, Haque et al. [47] reported 0.69 and 0.73 values for PIC and Nei coefficients for SSR markers in wheat genotypes, respectively. The SSR and ISSR markers had successfully been applied in barley [22] and D genome of *Triticum* and *Aegilops* species [48].

In this research, once we applied the information of both markers together, the results of AMOVA showed that the level of the genetic diversity for species (74.1), genome (85.7), and ploidy levels (91.1) of examined accessions were more than the situation that the genetic diversity was estimated for each marker alone. It means both markers’ information could result in a better understanding and a more significant genetic diversity among accessions of the different species, once studying different genomes and ploidy levels. The achieved results correspond to the previous studies, which had reported high levels of diversity in various species of *Aegilops* and *Triticum* through different DNA markers [14, 33, 47–49]. High values of Fst estimates reflected high proportion of genetic differentiations among the populations investigated. Similar effective differentiation based on SSR data had been reported by Yu et al. [50] in *Aegilops tauschii.* Also, high values of Fst estimated here also confirmed significant proportion of genetic diversity among populations in the AMOVA analysis.

According to the results based on Shannon information index, number of effective alleles, and polymorphism percentage indices with values of 0.46, 1.51, and 85%, respectively the highest amounts of genetic diversity was found for *aestivum* species while using two markers at the same time. Likewise, the ABD genome and 2n=4X=28 ploidy level showed the highest genetic diversity bast on above mentioned indices. Moreover, the lowest amount of diversity was for *neglecta* specie, U genome, and 2n=2X=14 ploidy level. After the *aestivum* species, species of *tauschii* and *boeoticum* had the most genetic diversity. The highest variability observed for ABD genome was due to the richness of allelic variation in the aestivum species whereas the highest variability in tetraploids here was due higher number of 4X accessions as compared to other ploidy level studied in this research. The same reason for the lowest genetic variability showed by neglecta can be addressed to the lowest number of accessions studied in this species. Etminan et al. [19] reported the most observed diversity for *aestivum* species, followed by Ae. *crassa* and *Ae. cylindrical.* The high level of genomic diversity in *Aegilops* and *Triticum* species can act as an ideal genetic repository to discover novel genes for modern wheat cultivars [49].

Based on the coordinate analysis, the *boeticum*, *aestivum*, and *triuncialis* species had high within population genetic diversity. Therefore, screening within these populations for abiotic stresses may lead to select promising accession to utilize in wheat improvement programs, while utilizing the species with low genetic diversity could be misleading. According to the coordinate analysis, the primers applied were more effective in separation the *boeoticum* species than other species. However, Khodaee et al. [33] reported the Iranian accessions of the genetic grouping pattern of *Aegilops triuncialis* based on the SCoT marker and concluded The differences between markers and primers, primers sequence, or the sample size might cause different results in different studies. Also, principal coordinates analysis revealed that *aestivum* species (ABD genome) was neighbor and had overlap with other species indicating gene introgression from wild relatives to bread wheat. Therefore, these relatives had high potential to improve new wheat cultivars for adverse environmental conditions.

Structure analysis based on SSR marker data could highly be effective and clearly explain an existing genetic variation among populations [51]. Structure analysis approved PCoA grouping results and A, D, and U genomes separated. This indicates that primers used were genome-specific and could be used in further genome explanatory studies.

## Conclusion

In the present study, the molecular results proved the existence of genetic diversity between the studied accessions. Molecular results explained the similarities and differences in the population genetics of the relatives of the National Plant Gene Bank of Iran and led to the identification of polymorphic bands. As a result, these polymorphic bands showed significant correlations with phenotypic grouping. These markers can be utilized to screen wheat genotypes for different traits. Primers used in the present study were effective and genome-specific and could be used in further genome explanatory studies.

## Data Availability

All data generated or analyzed during this study are included in this published article.

## Abbreviations

SSR: Simple Sequence Repeats
ISSR: Inter-simple sequence repeats
MI: Marker index
PCoA: Principal coordinate analysis
RP: Resolving power
